# DECONSTRUCTING MULTIMORBIDITY AND MUSCLE MITOCHONDRIAL RESPIRATION AND ENERGETICS IN OLDER ADULTS

**DOI:** 10.1093/geroni/igad104.2084

**Published:** 2023-12-21

**Authors:** Theresa Mau, Sofhia Ramos, Philip Kramer, Anne Newman, Steven Cummings, Russell Hepple, Paul Coen, Peggy Cawthon

**Affiliations:** San Francisco Coordinating Center, San Francisco, California, United States; AdventHealth, Orlando, Florida, United States; Wake Forest University School of Medicine, Winston-Salem, North Carolina, United States; University, Pittburgh, Pennsylvania, United States; California Pacific Medical Center Research Institute, San Francisco, California, United States; University of Florida, Gainesville, Florida, United States; AdventHealth Research Institute, Orlando, Florida, United States; California Pacific Medical Center, Research Institute, San Francisco, California, United States

## Abstract

Lower cardiorespiratory fitness among older adults is associated with greater multimorbidity burden, including cardiovascular disease, cancer, and dementia. Those with poorer muscle mitochondrial energetics also have lower cardiorespiratory fitness. We have previously shown lower mitochondrial respiration and energetics (OXPHOS and ATPmax) have higher morbidity burden using a summary measure, but the associations among specific conditions have not been examined. We leverage data from the SOMMA cohort (N=856, 76.4yrs, 59% women, 85.5% white); participants self-reported physician diagnosis of 11 age-related conditions (i.e., cancer, cardiac arrhythmia, chronic kidney disease (CKD), chronic obstructive pulmonary disease, coronary heart disease (CHD), depression, diabetes). Mitochondrial respiration was assessed by high-resolution respirometry of vastus lateralis using permeabilized fiber bundles. 31P magnetic resonance spectroscopy of the quadriceps measured ATPmax. In adjusted logistic models with beta-coefficients reported per SD decrement in each mitochondrial measure, fatty-acid-supported Leak respiration was associated with CKD (OR: 2.3(95%CI=1.2,4.3)), fatty acid and complex I&II supported oxidative phosphorylation (OXPHOS) with depression (OR: 1.8(95%CI=1.1,2.7)). Many respirometry measures and ATPmax associated with diabetes, with maximal electron transport system (ETS) capacity having the highest odds (OR: 1.5(95%CI=1.1,2.0)). No other associations were statistically significant for conditions listed above. These results indicate that mitochondrial respiration and energetics are more impaired in depression, kidney disease, and diabetes. This raises new questions about the role of mitochondrial respiration and energetics as cause or consequences of these conditions. Overall, our analyses suggest compromised mitochondrial function may have implications for specific aging-related conditions, and global measures of morbidity burden may not reflect these distinctions.

